# Lineage tracing reveals KSP^+^ cells as multipotent progenitors driving postnatal renal development and injury repair via notch signaling

**DOI:** 10.3389/fcell.2026.1893353

**Published:** 2026-09-09

**Authors:** Xinghao Duan, Fuyu Ji, Haoyu Zhu, Ruoqi Hu, Caiyue Li, Wenbo Ma, Ping Zhao, Yongbo Peng, Jinhua Shen, Guanghui Yu, Chao Gao

**Affiliations:** 1 College of Life Sciences, South-Central Minzu University, Wuhan, Hubei, China; 2 The Eighth Affiliated Hospital of Southern Medical University, Foshan, Guangdong, China; 3 Hubei Provincial Key Laboratory for Protection and Application of Special Plants in Wuling Area of China, College of Life Sciences, South-Central Minzu University, Wuhan, Hubei, China

**Keywords:** kidney development and repair, KSP^+^ cell, lineage tracing, notch signaling, progenitor cells

## Abstract

**Background:**

Progenitor cells generate multiple cells during development and are thought to be involved in tissue repair. The widespread distribution of Kidney-specific cadherin-positive (KSP^+^) cells in the kidney suggests they play an important role in renal development. However, direct evidence demonstrating that KSP^+^ cells function as progenitor cells contributing to renal development and repair is lacking.

**Methods:**

To obtain direct evidence for the differentiation and regenerative potential of KSP^+^ cells, we performed *in vivo* lineage tracing using *Ksp-Cre* and *Rosa-LSL-tdTomato* (*RFP* ^*f/f*^) transgenic mice, combined with thymidine analog (5-chloro-2′-deoxyuridine, CldU) labeling in adult wild-type (WT) mice. Lineage tracing was analyzed from embryonic day 14.5 (E14.5) to adulthood by triple immunofluorescence staining for KSP, red fluorescent protein (RFP), and nephron segment-specific markers. Unilateral ureteral obstruction (UUO) was performed to investigate the regenerative potential of KSP^+^ cells following renal injury. Proliferative capacity was assessed by CldU/Ki67/PCNA labeling from days 2–14 post-surgery. To explore the molecular mechanism potentially underlying KSP^+^ cell-mediated repair, we analyzed Notch signaling pathway in UUO-subjected WT mice.

**Results:**

The *Ksp-Cre: RFP* ^*f/f*^ lineage-tracing model exhibited high efficiency and specificity, with RFP expression strictly confined to KSP^+^ cells at E14.5. Lineage tracing revealed that embryonic KSP^*+*^ cells function as progenitor cells, giving rise to cells in the formation of multiple nephron segments, including proximal tubules (PTs), loops of Henle (HLs), distal convoluted tubules (DCTs), and collecting ducts (CDs) during kidney development. Comparison of the differentiation capacity of KSP^+^ cells during embryonic versus postnatal development indicated that KSP^+^ cells preferentially contribute to postnatal kidney development. In adult kidneys under homeostatic conditions, KSP^*+*^ cells showed limited proliferative potential. However, following UUO injury, KSP^+^ cells exhibited markedly increased proliferation. Notably, the proliferative response of KSP^+^ cells was closely correlated with Notch activation.

**Conclusion:**

Our lineage tracing data supports the KSP^+^ lineage behaves as a multipotent progenitor population. These cells give rise to cells of multiple nephron segments and preferentially drive postnatal kidney development. In adult kidneys, KSP^+^ cells are quiescent under homeostasis but become highly proliferative after renal injury, and this proliferative response is closely associated with activation of the Notch signaling pathway.

## 1 Introduction

Chronic kidney disease (CKD) has emerged as a global public health challenge, with irreversible renal fibrosis representing the common end point of its pathological progression. Acute renal injury often progresses to irreversible CKD due to defective endogenous repair. Therefore, identifying resident renal progenitors is critical for developing novel strategies to block CKD progression (Kellum et al., 2021; Kandel et al., 2025). Although the kidney possesses intrinsic regenerative capacity following acute injury, the prevailing understanding suggests that this repair mechanism relies primarily on the dedifferentiation and proliferation of surviving renal tubular epithelial cells (Li et al., 2024). However, under persistent or repetitive injury stimuli, this repair paradigm, dominated by mature cells, often fails to fully restore tissue integrity, ultimately leading to progressive structural damage and functional decline (Xia et al., 2018). Therefore, identifying and characterizing resident progenitor cell populations with regenerative potential in the kidney is considered a pivotal strategy to overcome current therapeutic limitations.

Kidney-specific cadherin (KSP) is a tubular epithelial marker whose expression initiates at E14.5 during mouse metanephric development (Shao et al., 2002a), and is widely expressed in all nephron segments in adult kidneys (Thedieck et al., 2005). The relatively late onset of KSP expression in the developing metanephros suggests that KSP does not participate in the early renal morphogenesis. In mice, high KSP expression across all nephron segments suggests that this cadherin may maintain the phenotype of terminally differentiated tubular epithelial cells, as previously proposed (Igarashi, 2004). *Ksp*-deficient mice exhibit marked developmental delay and urinary concentrating defects after birth, suggesting that KSP^+^ cells contribute to late stages of kidney development, particularly in the morphogenesis of functional regions such as the collecting duct (Thomson et al., 2021). Given their late onset at E14.5 and the postnatal developmental defects observed in Ksp-deficient mice (Thomson et al., 2021), KSP^+^ cells are unlikely to be merely terminally differentiated cells, but may instead function as multipotent progenitors. However, direct evidence confirming their ability to generate distinct nephron segments has been lacking. Moreover, although renal cells remain quiescent in the adult kidney under homeostatic conditions, it is unclear whether KSP^+^ cells are activated in response to injury to repair damaged renal tubules.

Here, we report *in vivo* lineage tracing using constitutive *Ksp-Cre* transgenic mice in combination with thymidine analogue labeling in WT mice (Shao et al., 2002b; Wang et al., 2015). Our lineage tracing data indicates that the KSP^+^ lineage gives rise to all nephron tubule segments, from the proximal tubule to collecting duct, during kidney development, thereby confirming their multipotency. While previous studies have primarily described the renal-specific tissue distribution of KSP^+^ cells, investigations into renal progenitors have either been limited to *in vitro* experiments (Morizane et al., 2014) or restricted to *in vivo* tracking of specific distal segments (Gao et al., 2022), lacking comprehensive *in vivo* evidence to delineate their full developmental trajectory and broad multipotency. In contrast, our *in vivo* tracing indicates that the KSP^+^ lineage spans the entire nephron. In adult mice, although the majority of KSP^+^ cells remain quiescent, a subset functions as primary responders that rapidly proliferate following injury and participate in renal tubular repair. Notably, this reparative activation is closely associated with the upregulation of the Notch signaling pathway in the proliferative KSP^+^ cell population (Mukherjee et al., 2019a). Our study highlights that KSP^+^ cells function as renal progenitor cells capable of generating multiple nephron tubule segments during kidney development and reveals that adult KSP^+^ cells contribute to renal tubule repair in response to injury. All abbreviations are listed in Supplementary Table S1.

## 2 Methods

### 2.1 Reagents

The primary antibodies used were mouse anti-Cadherin-16 (Servicebio, GB15953), rabbit anti-RFP (Servicebio, GB115053), multiple mouse anti-RFP antibodies (Servicebio, GB125053, GB155053, GB125054), rabbit anti-Megalin/LRP2 (ABclonal, A24690), rabbit anti-Calbindin (ABclonal, A4284), rabbit anti-AQP1 (ABclonal, A4195), rabbit anti-AQP2 (Servicebio, GB112259), rabbit anti-Uromodulin (ABclonal, A24641), mouse anti-Brdu (ABclonal, A1482), rabbit anti-Ki67 (ABclonal,A23722), rabbit anti-PCNA (ABclonal,A22426), rabbit anti-HES1 (ABclonal, A0925), rabbit anti-Notch1 (ABclonal, A7636), rabbit anti-JAG2 (ABclonal, A14247), mouse anti-GAPDH (Servicebio, GB15002). The secondary antibodies from Jackson ImmunoResearch were Alexa Fluor 488-conjugated AffiniPure Fab Fragment donkey anti-rabbit IgG (Jackson, 172404), Alexa Fluor 594-conjugated AffiniPure Fab Fragment donkey anti-rabbit IgG (Jackson, 167372), Alexa Fluor 594-conjugated AffiniPure Fab Fragment donkey anti-mouse IgG (Jackson, 170316). The secondary antibodies from ABclonal were AB flo488 goat anti-mouse IgG (ABclonal, AS053), AB flo555 goat anti-rabbit IgG (ABclonal, AS058), AB flo594 goat anti-rabbit IgG (ABclonal, AS039), AB flo647 goat anti-mouse IgG (ABclonal, AS059). The secondary antibodies for Western blot were HRP-conjugated goat anti-mouse IgG (H + L) (ABclonal, AS003) and HRP-conjugated goat anti-rabbit IgG (Servicebio, GB23303). Chloro-deoxyuridine (HY-112669) was purchased from MCE.

## 3 Breeding and genotyping of *Ksp-Cre: RFP* ^*f/f*^ reporter mice

Both *Ksp-Cre* and *Rosa-LSL-tdTomato* (Ai14) are commercially available transgenic mouse lines. This *Ksp-Cre* line exhibits high cellular specificity and minimal off-target expression. *Ksp-Cre: RFP* ^*f/f*^ mice were obtained by crossing *Ksp-Cre* (Cyagen, C001452) mice with *Rosa-LSL-tdTomato* (Cyagen, C001476) mice. Genotyping was performed by PCR according to the manufacturer’s instructions. All mice were maintained on a pure C57BL/6J background. Both male and female mice were used at the age of 2–6 months with free access to water and a normal diet unless otherwise indicated.

### 3.1 Unilateral ureteral obstruction and chloro-deoxyuridine labeling

UUO surgery was performed as described (Zhang et al., 2020; Chi et al., 2017). In brief, mice were anesthetized with inhaled isoflurane, and the left ureter was ligated via a lateral abdominal incision. CldU stock solutions (20 mg/mL) were freshly prepared in sterile 0.9% saline by heating to 45 °C. A single intraperitoneal injection of CldU (50 mg/kg) was administered at 24 h post-UUO surgery. Sham-operated group also received intraperitoneal injection of CldU (50 mg/kg) 24 h postoperatively. Kidney tissues were then harvested at the indicated time points (D0, D2, D6, and D14). No repeated injections were performed. Kidney samples were harvested for analysis.

### 3.2 Western blotting

Kidney tissues were lysed using RIPA lysis buffer (Servicebio, G2002) to extract total protein. Protein samples were separated by 8%–10% SDS-PAGE and transferred onto PVDF membranes. After blocking with 5% skim milk for 1 h at room temperature, membranes were incubated with specific primary antibodies at 4 °C overnight. Membranes were then washed thoroughly with TBST and incubated with the corresponding HRP-conjugated secondary antibody for 1 h at room temperature. Signals were detected using an ECL chemiluminescent substrate kit (ABclonal, RM02867). Band intensities were quantified with ImageJ and normalized to GAPDH. Experiments were repeated at least three times.

### 3.3 Renal physiological parameter assessment and metabolic balance monitoring

All mice used were healthy, aged 2–3 months, with 6–8 mice (equal numbers of males and females) per group. Body weight, water intake, and food consumption were continuously recorded for at least 2 weeks. Urine sodium and potassium levels were measured using a flame photometer (Youke Instrument, FP6400). Non-invasive tail-cuff blood pressure measurement systems (Softron Biotechnology, BP-2010A) were used to measure systolic and diastolic blood pressure at a fixed time daily. Measurements were performed consecutively for 3 days, and the average value was calculated. Whole blood samples were collected via cardiac puncture or retro-orbital bleeding. Plasma urea nitrogen (Nanjing Jiancheng Bioengineering Institute, C000-2-1), creatinine (Nanjing Jiancheng Bioengineering Institute, C013-2-1), and other electrolyte concentrations were measured using an automated blood biochemistry analyzer (Seamaty Technology, VET-VG1).

### 3.4 Immunofluorescence studies

Kidneys were fixed in 4% paraformaldehyde overnight at 4 °C, embedded in paraffin, and cut into 4–5 μm sections for epifluorescence and confocal microscopy. After boiling in antigen retrieval buffer (0.01M sodium citrate, pH 6.0), paraffin sections were blocked with 5% BSA/0.5% Triton X-100 in PBS, followed by labeling with primary antibodies from different species in various combinations as detailed in each figure. Primary antibodies were diluted at 1:100-1:1000 in 1% BSA and applied to sections overnight at 4 °C. After 3 × 5-min washes in PBS, sections were incubated with appropriate combinations of secondary antibodies and mounted with anti-fade mounting medium (Servicebio, G1407). All sections were examined using an epifluorescence microscope (Zeiss, Axioscope 5) or a confocal microscope (Leica, Stellaris 5). For consistent visualization across figures, Adobe Photoshop CS4 was used solely to standardize display color channels across all panels; no contrast/brightness manipulations were applied that would alter signal intensity or quantitative marker co-localization results. Original channel colors were maintained without color substitution. Immunofluorescence analysis was performed by two or three independent investigators (X.D., F.J., and/or C.G.) on at least two sections per kidney from three or more mice per condition. All immunofluorescence experiments were independently repeated at least three times. Detailed antibody and fluorophore channel assignments for each marker are specified in the Reagents section. X.D. and F.J. were blinded to other aspects of the majority of experiments, while C.G. was not involved in the analysis of the remaining experimental sets. For CldU detection, an additional acid denaturation step (2 M HCl, 30 min at room temperature) was performed after antigen retrieval for epitope exposure, followed by incubation with an anti-BrdU antibody (cross-reactive with CldU, ABclonal A1482) at a 1:200 dilution overnight at 4 °C.

### 3.5 Cell counting and quantification of KSP^+^ cell-derived X^+^ cells

Cell counting was conducted as previously reported (Gao et al., 2022). To robustly identify structural boundaries and morphological features of each segment, we selected classical, well-validated surface markers widely used in published lineage tracing studies (Nielsen et al., 2016; Fisher and Howie, 2006; Mark et al., 1996; Nielsen and Agre, 1995; van Lieburg et al., 1995): Megalin^+^ or Aquaporin1^+^ (AQP1^+^) for proximal tubules (PTs), Uromodulin^+^ (Umod^+^) for thick ascending limb and early distal tubule cells, Calbindin^+^ for distal convoluted tubules (DCTs) and connecting tubules (CNTs), and Aquaporin2^+^ (AQP2^+^) for collecting ducts (CDs). A cell was scored as positive for a marker if its signal intensity exceeded the background signal from adjacent cells within or outside the same tubule. In each case, at least 200 tubules were analyzed, comprising at least 1500 cells from 4 mice, unless otherwise indicated. DAPI staining was used to aid counting. The percentage of KSP^+^ cell-derived X^+^ (X equals to specific nephron segment marker) cells was defined as the number of KSP^−^RFP^+^X^+^ cells divided by the total number of X^+^ cells (×100%), reflecting the differentiation or trans-differentiation of KSP^+^ cells into segment-specific cell types.

### 3.6 Statistical analyses

Details regarding replicate numbers for each experiment are provided in the respective figure legends. Specifically, all experiments utilized a minimum of 3 mice per group (n ≥ 3), with the exact sample size (n) for each quantification detailed in the corresponding figure legends. Unless otherwise indicated, all experiments were independently repeated at least three times. Unless otherwise indicated, data are pooled from or representative of three or four independent experiments and shown as mean ± SEM. Data distribution and normality were evaluated. Comparisons between groups were then assessed by Student’s t-test or one-way analysis of variance (ANOVA) for data meeting the assumptions of parametric tests. All statistical analyses were performed using GraphPad Prism software, with p < 0.05 considered statistically significant. Significance levels are denoted as follows: p < 0.05 (*), p < 0.01 (**), p < 0.001 (***), or ns (not significant).

## 4 Results

### 4.1 Generation of a new KSP-Lineage tracing mouse model

**FIGURE 1:**
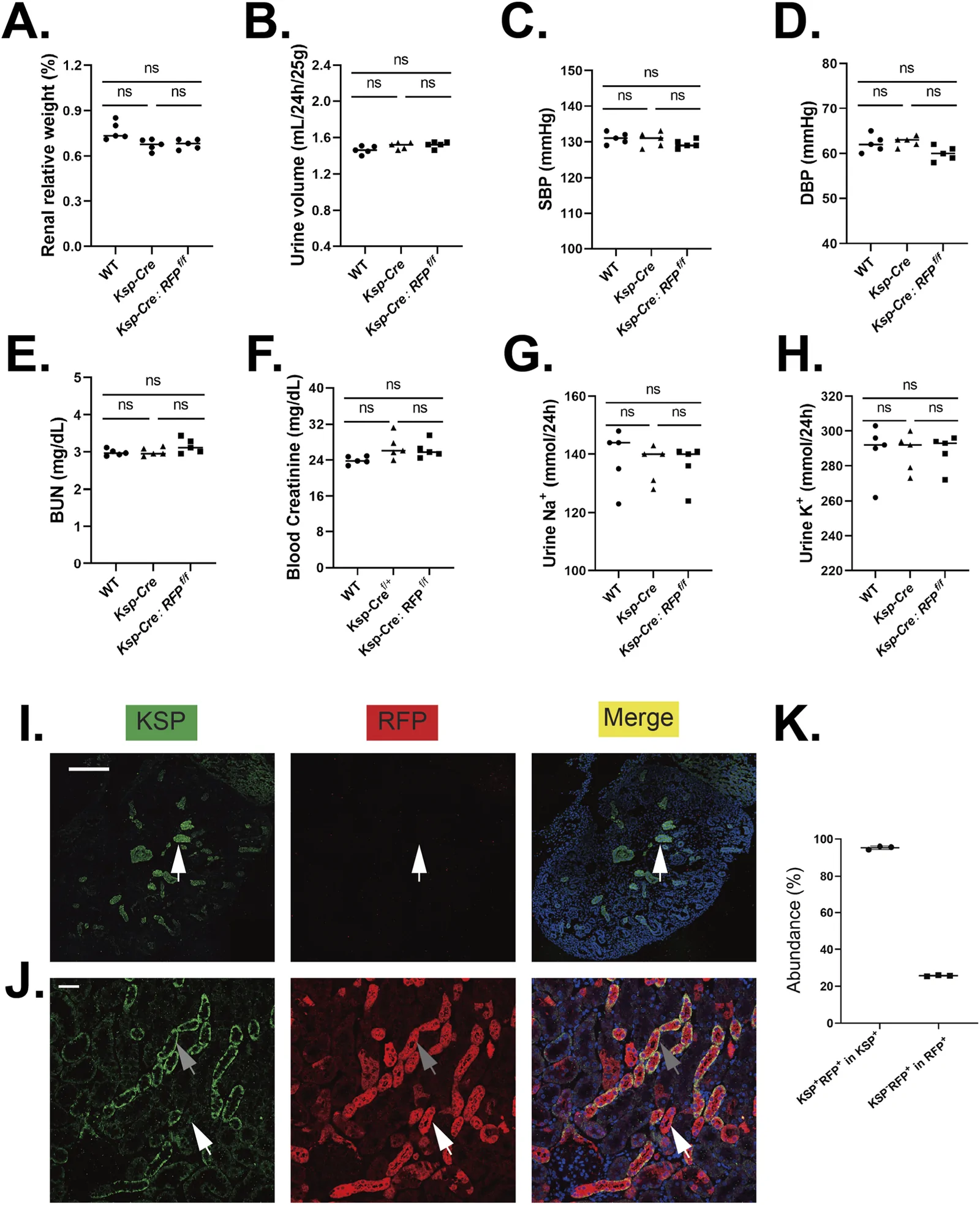
Generation and validation of a novel *Ksp-Cre: RFP* ^*f/f*^ lineage-tracing mouse model. **(A–H)** Physiological characterization of adult wild-type (WT), *Ksp-Cre*, and *Ksp-Cre: RFP* ^*f/f*^ mice. No significant differences were observed among genotypes in kidney-to-body weight ratio **(A)**, 24-h urine volume **(B)**, systolic blood pressure (SBP) **(C)**, diastolic blood pressure (DBP) **(D)**, blood urea nitrogen (BUN) **(E)**, serum creatinine **(F)**, urinary Na^+^ excretion **(G)**, or urinary K^+^ excretion **(H)**. Data are shown as individual values with mean ± SEM. n = 5 mice per group. I-K. Specificity and efficiency of RFP reporter expression in the *RFP* ^*KC*^ model. **(I)** In *RFP* ^*f/f*^ mice lacking Cre recombinase, endogenous KSP protein (green) was readily detected, whereas RFP signal (red) was completely absent. Arrowhead indicated representative KSP^+^RFP^−^ tubule. Scale bars, 80 µm. **(J)** In *RFP* ^*KC*^ mice, an overview of the corticomedullary junction showing robust RFP expression (red) extensively co-localized with KSP (green) in renal tubular cells. The lower-left region depicts the renal cortex (containing glomeruli), and the upper-right region depicts the outer medulla. Gray arrowheads indicate representative KSP^+^RFP^+^ tubules, and white arrowheads indicate representative KSP^−^RFP^+^ tubules. Scale bars, 50 µm. **(K)** Quantitative analysis of reporter specificity and efficiency in adult *RFP* ^*KC*^ mice. The percentage of KSP^+^RFP^−^ cells in all KSP^+^ cells was 5.52% ± 0.71% (n = 4). The percentage of KSP^−^RFP^+^ cells in all RFP^+^ cells was 23.55% ± 0.73% (n = 4). 300 tubules were quantified per mouse across 5 fields.

To trace the lineage fate of KSP^+^ cells *in vivo*, we crossed mice carrying the *Ksp-Cre* transgene with *Rosa-LSL-tdTomato* (commonly named Ai14) mice, which harbor a *loxP-STOP-loxP-tdTomato* cassette inserted into the *Rosa26* locus. The genotypes of double-transgenic mice (*Ksp-Cre: RFP* ^*f/f*^, *RFP* ^*KC*^) were verified by PCR using primers listed in Supplementary Table S2 (representative genotyping gel in Supplementary Figure S1). Adult *RFP* ^*KC*^ mice aged 3 months were used for subsequent studies. These mice grew and developed normally and exhibited no significant differences from WT or *Ksp-Cre* littermates in kidney-to-body weight ratio, urine volume, systolic blood pressure (SBP), diastolic blood pressure (DBP), blood urea nitrogen (BUN), serum creatinine, urinary Na^+^, or urinary K^+^ (Figures 1A–H), indicating that genetic manipulation did not overtly compromise renal physiological function. To rigorously validate whether RFP reporter expression in this model specifically depends on Cre recombinase activity, we performed immunofluorescence staining. In *RFP* ^*f/f*^ mice lacking Cre recombinase, although endogenous KSP protein was readily detectable, no RFP signal was observed in the whole kidney (Figure 1I). In contrast, the RFP reporter was robustly activated and extensively expressed throughout the kidney of *RFP* ^*KC*^ mice, as clearly visualized at the corticomedullary junction (Figure 1J). These results definitively confirmed that RFP expression was strictly dependent on Cre expression, excluding the possibility of reporter leakage. Further quantitative immunofluorescence co-localization analysis revealed that in the kidneys of *RFP* ^*KC*^ mice, approximately 5.52% ± 0.71% of KSP^+^ cells lacked RFP signal, likely due to incomplete Cre-mediated recombinase efficiency. Notably, a substantial fraction of RFP-expressing cells (23.55% ± 0.73%) lost KSP protein expression. While this suggests a potential shift in cellular identity, it is important to note that within a constitutive Cre system, this KSP^−^RFP^+^ phenotype could also reflect the downregulation of KSP in differentiated cells rather than a true change in cell fate (Figure 1K). Collectively, these findings indicate that RFP expression in the *RFP* ^*KC*^ mouse model was dependent on KSP promoter-driven Cre recombinase activity, enabling efficient and specific lineage tracing of KSP^+^ cells in the kidney.

### 4.2 *RFP* ^*KC*^ model enables faithful lineage tracing of KSP^+^ cells during kidney development

**FIGURE 2:**
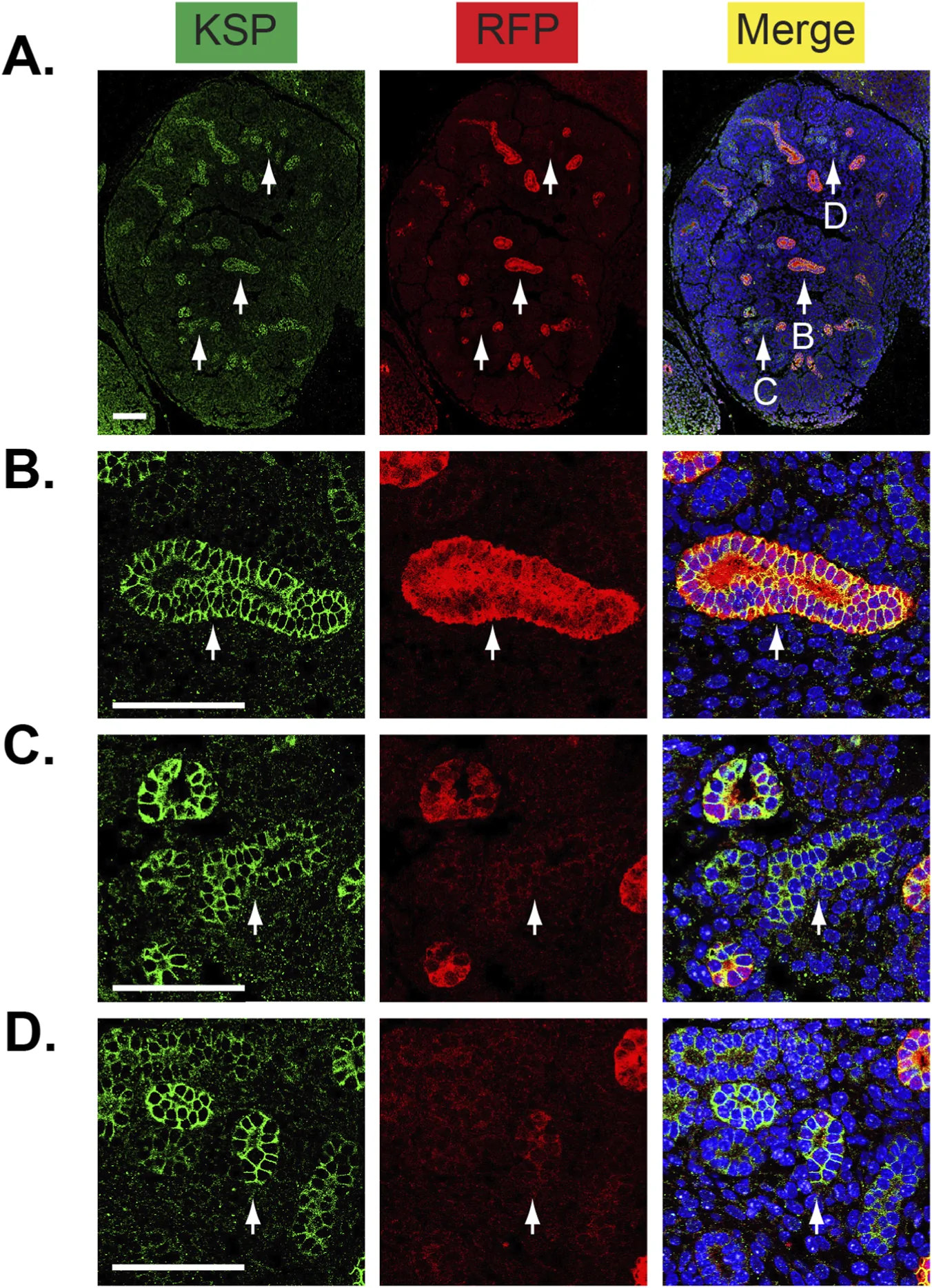
RFP expression was strictly confined to KSP^+^ cells at E14.5 in *RFP* ^*KC*^ mice. **(A)** Whole kidney overview of E14.5 *RFP* ^*KC*^ kidney showed extensive RFP expression (red). Although the green KSP signal is difficult to visualize at this low magnification, higher-magnification images **(B–D)** confirmed that all RFP+ cells co-expressed KSP. Arrows indicated representative tubules in **(B–D)**. Scale bar, 50 µm. **(B)** A representative tubule showed all cells were KSP^+^RFP^+^ cells (arrow). **(C)** A representative tubule showed all cells were KSP^+^RFP^−^ cells (arrow). **(D)** A representative tubule showed a subset of KSP^+^ cells were RFP^−^ cells (arrow). Scale bars, 200 μm. n = 4, 5 fields were analyzed per mouse.

The expression of the RFP reporter in *RFP* ^*KC*^ mice is strictly dependent on Cre recombinase activity driven by the *Ksp* promoter. However, we observed a substantial population of RFP^+^ cells lost KSP expression in adult *RFP* ^*KC*^ mice (Figures 1J,K). To investigate the developmental origin of these KSP^−^RFP^+^ cells, we traced RFP expression throughout kidney development in *RFP* ^*KC*^ mice. Given that *Ksp* initiates expression in the kidney at E14.5 (Bray and Bigas, 2025; He et al., 2017; Santoro and Schultz, 2002), we confirmed KSP and RFP expressions from E10.5 to E16.5 by immunofluorescence staining and found KSP and RFP first appeared at E14.5 (Supplementary Figure S2). We examined RFP expression at E14.5, and found that all RFP-expressing cells were exclusively KSP^+^ (Figures 2A–D), indicating that the *RFP* ^*KC*^ model enables faithful lineage tracing of KSP^+^ cells during kidney development. Due to the inherent limitations of Cre-mediated recombination efficiency (Santoro and Schultz, 2002), a fraction of KSP^+^ cells at E14.5 lacked detectable RFP signal (Figures 2C,D). Collectively, these results indicate that at E14.5, RFP expression is driven by *Ksp* promoter and strictly confined to KSP^+^ cells in *RFP* ^*KC*^ mice, establishing that RFP serves as a faithful reporter for lineage tracing of KSP^+^ cell progeny throughout kidney development.

### 4.3 KSP^*+*^ cells serve as progenitor cells contributing to kidney maturation

**FIGURE 3:**
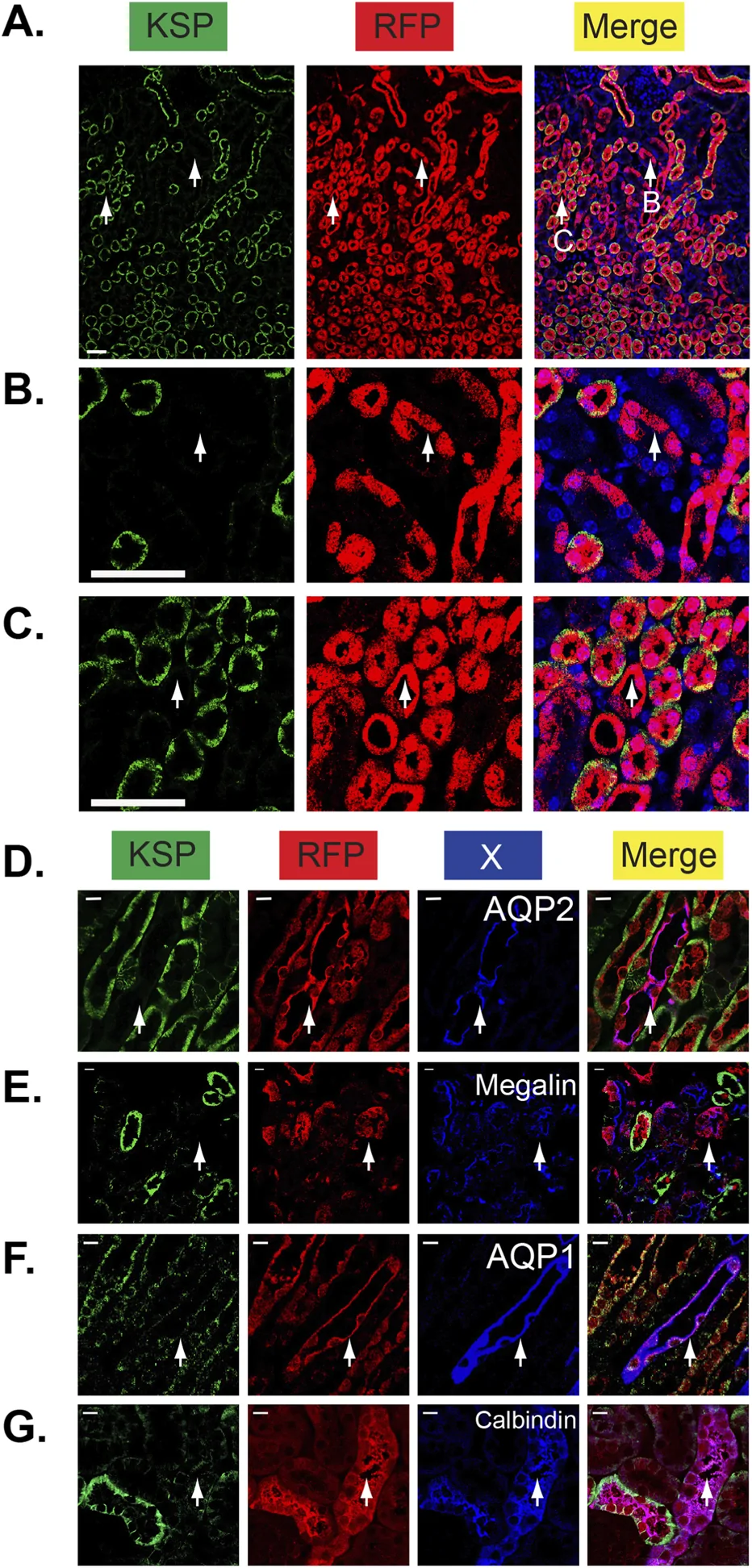
Embryonic KSP^+^ cells differentiate into epithelial cells of multiple major nephron segments in adult kidney at P90. **(A)** An overview of P90 kidney showing the extensive distribution of KSP (green) and RFP (red). Arrows indicate the representative regions magnified in **(B,C)**. **(B,C)** Magnified views of the boxed regions in A showing the typical KSP^−^RFP^+^ phenotype. Scale bars, 50 µm **(A)**; 200 µm **(B,C)** Triple immunofluorescence staining of kidneys from P90 *RFP* ^*KC*^ mice showing RFP-derived cells (red) that lost KSP expression (green) and gained segment-specific markers expression (blue). **(D)** AQP2^+^ collecting duct cells with KSP^−^RFP^+^ phenotype (arrow). **(E)** Megalin^+^ proximal tubule cells with KSP^−^RFP^+^ phenotype (arrow). **(F)** AQP1^+^ proximal tubule and descending thin limb cells with KSP^−^RFP^+^ phenotype (arrow). **(G)** Calbindin^+^ distal convoluted tubule cells with KSP^−^RFP^+^ phenotype (arrow). Scale bars, 30 µm **(D,F,G)**; 20 µm **(E)** n = 4. 200 tubules were quantified per mouse across 5 fields.

The presence of KSP^−^RFP^+^ cells in adult *RFP* ^*KC*^ mice aged 3 months (postnatal day 90, P90) (Figures 1J,K) suggests that these cells are derived from KSP^+^RFP^+^ cells present at E14.5, implicating KSP^+^RFP^+^ cells as early renal developmental progenitors. Low-magnification overviews initially confirmed that these KSP^−^RFP^+^ cells were not restricted to isolated foci, but were extensively distributed throughout the renal parenchyma (Figures 3A–C). To characterize the identity of these KSP^−^RFP^+^ cells, we performed triple immunofluorescence staining using nephron segment-specific markers spanning from PTs to CDs. We identified that a subset of Aqp2^+^ collecting duct cells expressed RFP (20.86%, Supplementary Table S7) but not KSP (Figure 3D). Similarly, a subset of Megalin^+^ proximal tubule cells (8.26%, Supplementary Table S3) expressed RFP without KSP (Figure 3E). The percentage of KSP^−^AQP1^+^RFP^+^ cells among all AQP1^+^ cells (proximal tubule and descending thin limb of the loop of Henle) was approximately 9.76% (Supplementary Table S4, Figure 3F), the percentage of KSP^−^Umod^+^RFP^+^ cells in all Umod^+^ cells (loops of Henle) was approximately 9.15% (Supplementary Table S5), and the percentage of KSP^−^Calbindin^+^RFP^+^ cells among all Calbindin^+^ cells (distal convoluted tubule) was approximately 15.96% (Supplementary Table S6, Figure 3G). These findings indicate that embryonic KSP^+^ cells substantially contribute to the development and maturation of adult renal tubules. Taken together, these lineage-tracing results indicate that embryonic KSP^+^ cells function as progenitor cells during kidney development, differentiating into epithelial cells of multiple major nephron segments in the adult mouse kidney.

### 4.4 KSP^+^ cells preferentially contribute to postnatal kidney development

**FIGURE 4:**
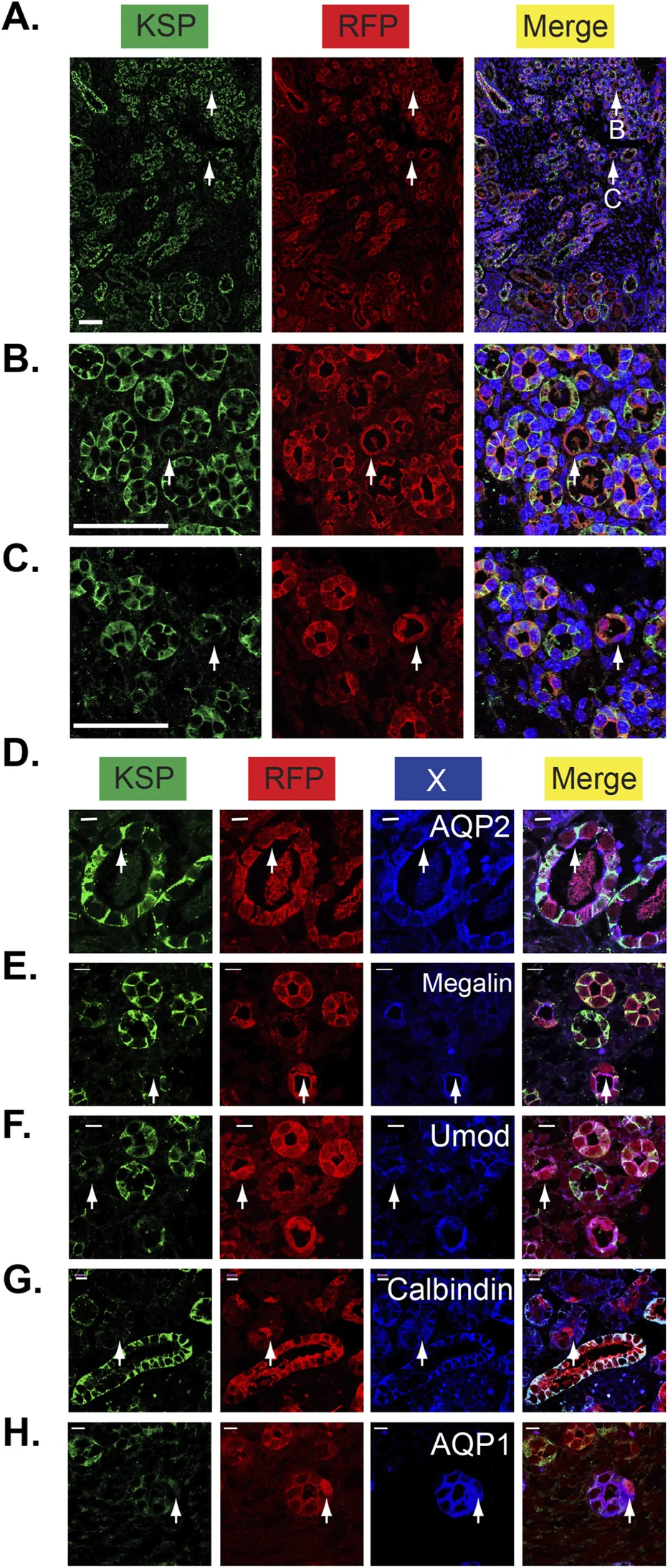
Lineage tracing of KSP^+^ cells at E19.5 in *RFP* ^*KC*^ mice. **(A)** An overview of E19.5 kidney showing extensive expression of KSP (green) and RFP (red) in developing nephrons. DAPI (blue) marks nuclei. Arrows indicated representative tubules in **(B,C)**. **(B)** a representative RFP^+^ tubule lost the expression of KSP. **(C)** a RFP^+^ cell lost the expression of KSP in a KSP^+^ tubule. Scale bars, 50 µm **(A)**; 200 µm **(B,C)**. **(D–H)** Triple immunofluorescence staining showing KSP^−^RFP^+^ cells gained the expression of different segment-specific markers at E19.5. **(D)** AQP2 for collecting duct cells (arrow). **(E)** Megalin for proximal tubule cells (arrow). **(F)** Umod for thick ascending limb cells (arrow). **(G)** Calbindin for distal convoluted tubule cells (arrow). **(H)** AQP1 for proximal tubule/descending thin limb cells (arrow). Scale bars, 30 µm **(D–F,H)**; 20 µm **(G)** n = 4. 200 tubules were quantified per mouse across 5 fields.

To determine whether KSP^+^ cells contribute to nephron formation at distinct stages of kidney development, we performed lineage tracing of KSP^+^ cells across multiple developmental time points. Examination of *RFP* ^*KC*^ kidneys at E19.5, P1, P30, P90, and P180 revealed a subset of RFP^+^ cells or tubules that had lost KSP expression at all stages analyzed. These KSP^−^RFP^+^ cells or tubules were broadly distributed across major nephron segments, including PTs (marked by Megalin or AQP1), HLs (AQP1 marking the descending thin limb and Umod marking the thick ascending limb), DCTs (marked by Calbindin), and CDs (marked by AQP2) (Figures 3, 4, Supplementary Figures S3-S5). We compared the proportions of KSP^−^X^+^RFP^+^ (X equals to specific nephron segment marker) cells among all X^+^ cells at different developmental stages and found that these proportions were very low at E19.5 (1.51%–4.41%, Supplementary Tables S3 to S7), with only occasional KSP^−^X^+^RFP^+^ cells observed within X^+^ tubules (Figure 4). As kidneys entered postnatal developmental, this proportions increased rapidly (1.98%–23.12%, Supplementary Tables S3 to S7), and multiple KSP^−^X^+^RFP^+^ cells became detectable within X^+^ tubules; in some cases, entire X^+^ tubules were composed of KSP^−^X^+^RFP^+^ cells (Figure 3, Supplementary Figures S3-S5). This trend indicates that KSP^+^ cells preferentially contribute to postnatal development. Collectively, these lineage-tracing results establish that KSP^+^ cells emerging at E14.5 function as renal progenitor cells during kidney development. At successive developmental stages, these KSP^+^ cells exhibit multipotent differentiation capacity, giving rise to mature epithelial cells across diverse nephron segments, including PTs, HLs, DCTs, and CDs.

### 4.5 Adult KSP^+^ cells are among the first responders to UUO-Induced injury

**FIGURE 5:**
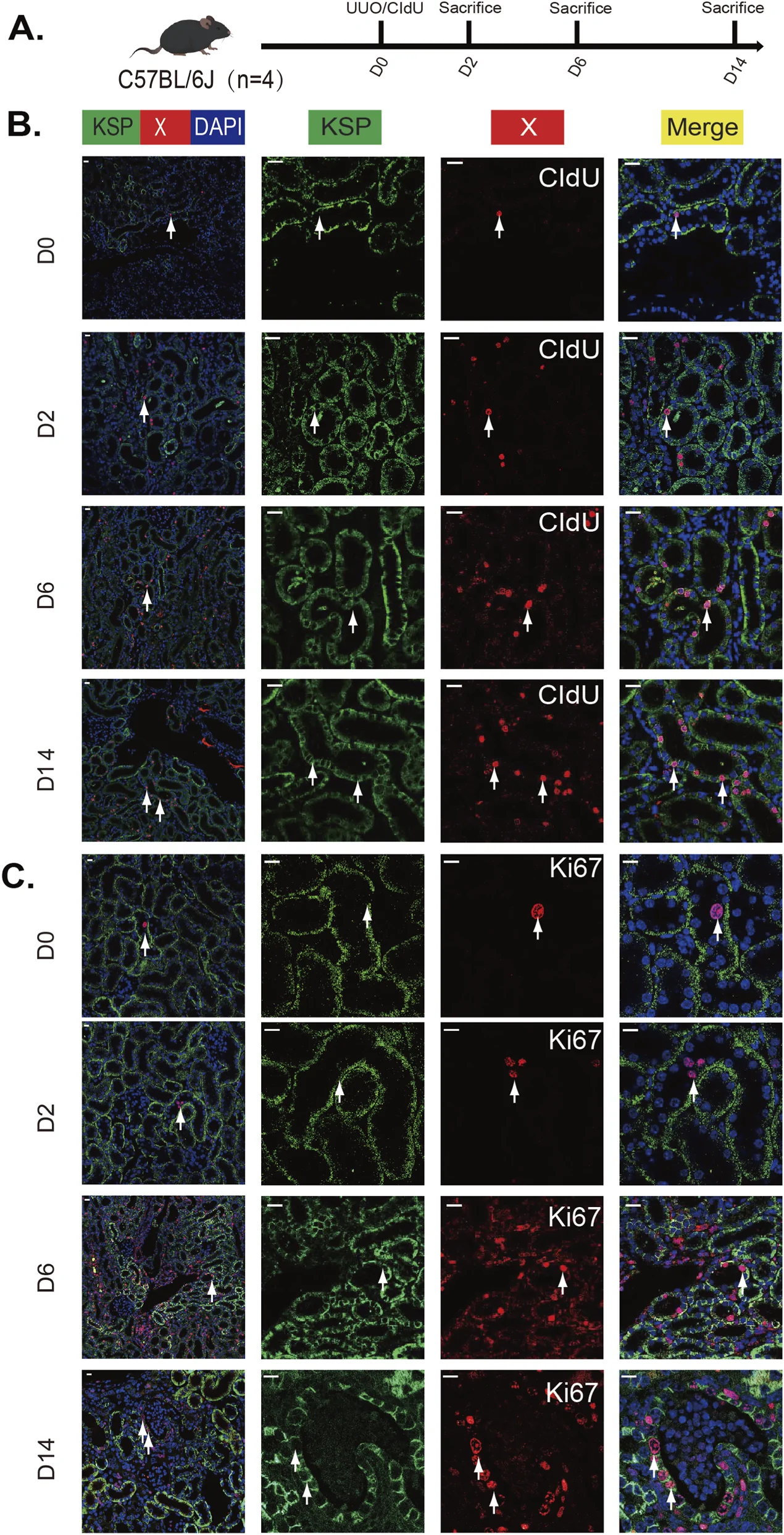
Temporal analysis of KSP^+^ cells proliferation following UUO-induced renal injury. **(A)** Experimental scheme showing UUO surgery with CldU administration and tissue harvest at D0 (sham), D2, D6, and D14. Each time point included n = 4 mice. **(B,C)** A low-magnification overview (left panel) is provided to show the overall distribution of CldU^+^/Ki67^+^ cells within the kidney tissue. **(B)** Representative immunofluorescence images of CldU^+^KSP^+^ cells at D0, D2, D6, and D14 post-UUO. Arrows indicate CldU^+^KSP^+^ cells. Scale bars, 40 µm. **(C)** Representative immunofluorescence images of Ki67^+^KSP^+^ cells at D0, D2, D6, and D14 post-UUO. Arrows indicate Ki67^+^KSP^+^ cells. Scale bars, 40 µm. 300 tubules were quantified per mouse across 5 fields.

To evaluate whether KSP^+^ cells in the adult kidney participate in injury repair, we subjected WT mice to UUO surgery with concurrent CldU administration and harvested kidneys at day 2, 6, and 14 post-injury (D2, D6, D14), with sham-operated mice as controls (Figure 5A). In sham-operated mice (D0), CldU^+^KSP^+^ cells accounted for only 0.05% (Supplementary Table S8) of total KSP^+^ cells, indicating that the vast majority of KSP^+^ cells remain quiescent under homeostatic conditions. Following UUO injury, this proportion increased markedly, reaching 0.06% at D2, 2.17% at D6, and further escalating to 6.83% by D14 (Figure 5B, Supplementary Table S8). To independently verify proliferative activation, we examined expression of the cell cycle markers Ki67 and PCNA. Ki67 labels cells in active cell cycle phases (G1/S/G2/M), whereas PCNA indicates ongoing DNA replication or DNA damage repair. In sham-operated kidneys, KSP^+^ cells maintained a quiescent state, with negligible fractions expressing Ki67^+^ (0.31%, Supplementary Table S10) or PCNA^+^ (0.25%, Supplementary Table S11). In contrast, the proportions of Ki67^+^KSP^+^ and PCNA^+^KSP^+^ cells increased as early as D2 (0.33% and 0.27%, respectively), with progressive elevation through D14 (12.61% and 12.87%, respectively) (Figure 5C, Supplementary Figure S6, Supplementary Tables S10-S11). Furthermore, quantification revealed the dynamic distribution of KSP-derived RFP^+^ cells across distinct nephron segments following UUO injury. During the early injury phase (D2 and D6), the proportion of KSP-derived cells among proximal tubule cells (marked by AQP1 and Megalin), thick ascending limb cells (Umod^+^), and collecting duct cells (AQP2^+^) generally increased, indicating their active participation in early reparative responses. However, by D14, likely due to severe injury progression, this proportion exhibited divergent trends: it declined in AQP1^+^ proximal tubules (14.76%) and AQP2^+^ collecting ducts (8.06%), whereas it continued to rise in Megalin^+^ proximal tubules (19.87%) and Umod^+^ thick ascending limbs (28.31%) (Supplementary Figures S7-S10, Supplementary Tables S12-S15). These results indicate that KSP^+^ cells re-enter the cell cycle and actively engage in DNA synthesis following injury. Thus, adult KSP^+^ cells, which remain quiescent under physiological conditions, are rapidly activated and initiate proliferation upon UUO-induced renal injury, positioning this population among the earliest key responders to injury, although a direct causal relationship between Notch signaling and this proliferative response remains to be functionally validated. While various cell types (such as interstitial cells) undergo proliferation following UUO injury, our CldU labeling indicated that KSP^+^ renal tubular epithelial cells were highly active and contributed substantially to this proliferative response. Although our earlier developmental data confirmed that KSP^+^ cells span all nephron segments, distinguishing the proliferative contributions of KSP^+^ cells among specific segments during injury response represents a key direction for future studies using segment-specific Cre tools.

### 4.6 Notch activation is closely associated with the functional activation of KSP^+^ cells after renal injury

**FIGURE 6:**
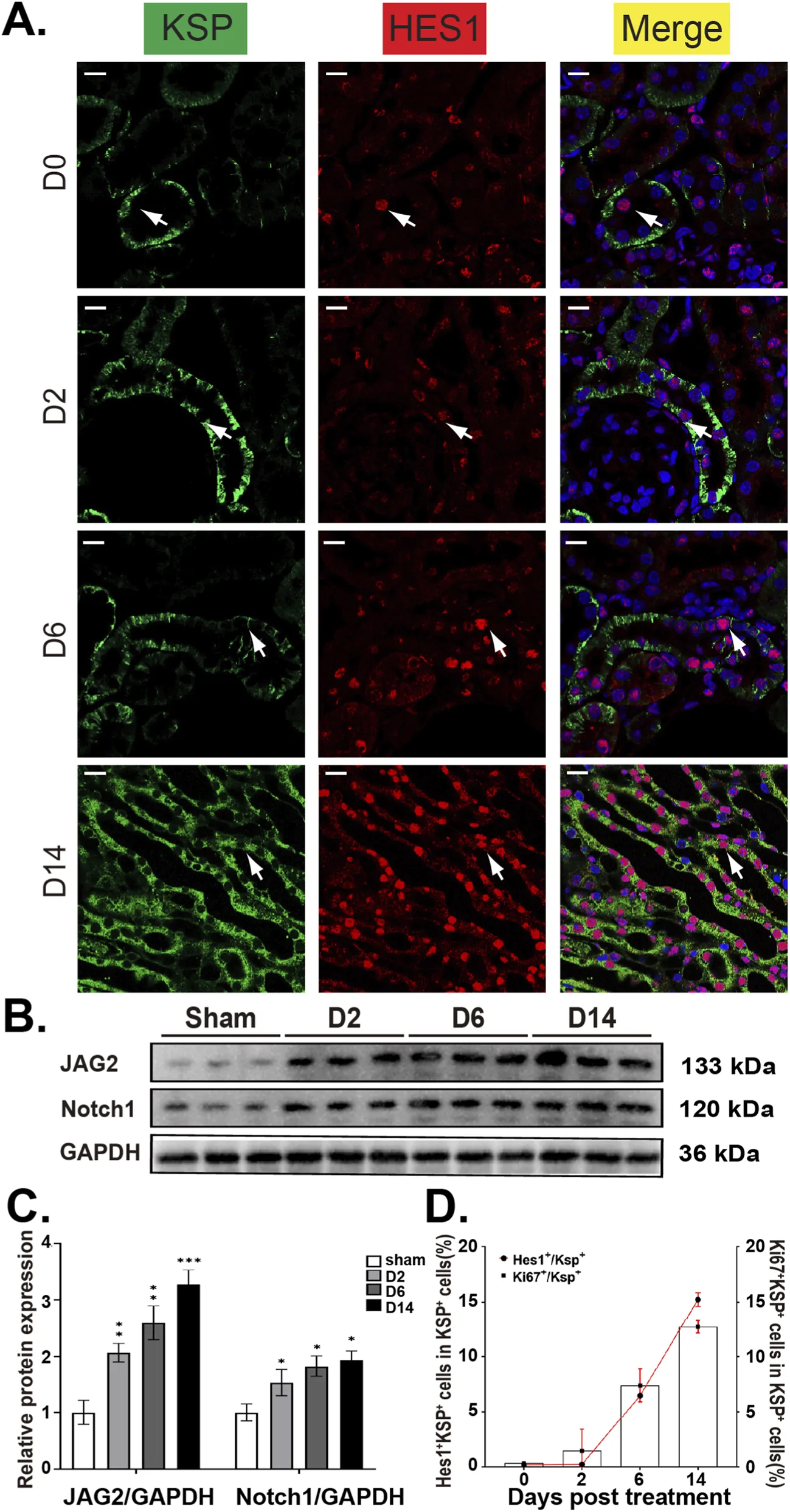
Activation of Notch signaling following UUO-induced renal injury. **(A)** Representative immunofluorescence images showing KSP (green) and Hes1 (red) co-expression across D0 (Sham), D2, D6, and D14. Arrows indicate Hes1^+^KSP^+^ cells. Quantification shows progressive increase of Hes1^+^KSP^+^ cells in all KSP^+^ cells **(D)**. 300 tubules were quantified per mouse across 5 fields. **(B)** Western blot analysis of JAG2 and Notch1 protein expression. **(C)** Relative quantification of JAG2 and Notch1 protein levels normalized to GAPDH. Data represent mean ± SEM from three independent experiments. **(D)** Correlation analysis between KSP^+^ cell proliferative index (percentage of Ki67^+^KSP^+^ cells in all KSP^+^ cells) and Notch activation index (percentage of Hes1^+^KSP^+^ cells in all KSP^+^ cells) across D0-D14 time points. n = 4.

Having established that KSP^+^ cells are rapidly activated following UUO injury, and considering that Notch signaling is a well-documented response in UUO models (Gao et al., 2022; Huang et al., 2018), we hypothesized that this pathway might underlie the injury-specific activation of KSP^+^ cells. To test this hypothesis, we systematically analyzed the dynamic activation of Notch signaling after renal injury and its correlation with the proliferative status of KSP^+^ cells. At the protein level, Western blot analysis revealed that Notch1 and its ligand JAG2 were expressed at low levels under homeostatic conditions. Following UUO injury, protein levels of both Notch1 and JAG2 increased significantly from D6 onward, with progressive elevation through D14 (Figures 6B,C). The Notch downstream transcriptional effector hairy and enhancer of split 1 (Hes1) exhibited a parallel expression pattern. In uninjured kidneys, only a small fraction of KSP^+^ cells expressed Hes1 (0.21%). This proportion increased markedly following UUO, rising to 0.25% at D2, 6.34% at D6, and reaching 14.55% by D14 (Figure 6A, Supplementary Table S9). This expression profile closely mirrored the proliferative dynamics (Ki67) of KSP^+^ cells (Figure 6D), suggesting a close association between the Notch-Hes1 axis and cellular proliferation. Collectively, these results indicate that Notch signaling is robustly activated in the adult mouse kidneys following injury, with its temporal dynamics highly synchronized with the proliferative activation of KSP^+^ cells. These findings implicate Notch pathway activation as being closely associated with the functional activation of KSP^+^ cells and potentially serving as a putative upstream regulatory mechanism.

## 5 Discussion

In this study, we employed a well-established standard *Ksp-Cre: RFP* ^*f/f*^ (*RFP* ^*KC*^) dual-transgenic lineage tracing system to analyze the fate of KSP^+^ cells in renal development. Validation of this system demonstrated that RFP expression is strictly dependent on Cre recombinase activity, with no background leakage detected in *RFP* ^*f/f*^ control mice (Figure 1I). Approximately 5% of KSP^+^ cells remained unlabeled by RFP (Figure 1K), consistent with documented variations in Cre recombinase efficiency and transgenic mosaicism (He et al., 2017; Santoro and Schultz, 2002). Notably, abundant KSP^−^RFP^+^ cells were observed in adult kidneys at P90 (Figure 1J), foreshadowing the differentiation potential of the KSP^+^ lineage and providing critical clues for subsequent tracing studies. Mechanistically, the 5.52% KSP^+^RFP^−^ subpopulation is likely attributable to inherent mosaicism in Cre recombination and variable Ksp promoter activity during early development (He et al., 2017; Santoro and Schultz, 2002). Conversely, the emergence of the 23.55% KSP^−^RFP^+^ population primarily reflects the transcriptional silencing of Cdh16 (KSP) as embryonic progenitors terminally differentiate into mature epithelial subtypes (Thedieck et al., 2005; Igarashi, 2004), a phenomenon that may be further influenced by differential antibody epitope accessibility in adult tissues. Consequently, we acknowledge that due to incomplete recombination efficiency, the quantitative estimates of KSP lineage contribution presented in this study represent conservative, relative proportions rather than absolute lineage outputs.

To test the differentiation potential of KSP^+^ cells, embryonic lineage tracing was performed to uncover their developmental origin. At E14.5, when KSP expression initiates in the kidney, all RFP^+^ cells were KSP^+^ (Figure 2). This finding confirmed that RFP labeling accurately reflects endogenous KSP expression and established E14.5 as an appropriate starting point for tracking KSP^+^ cell descendants. As development proceeded to the adult stage at P90, RFP^+^ progeny cells and tubules were extensively distributed throughout proximal tubules (Megalin^+^/AQP1^+^), Loops of Henle (Umod^+^/AQP1^+^), distal convoluted tubules (Calbindin^+^), and collecting ducts (AQP2^+^), while having lost KSP expression (Figure 3). These KSP^−^RFP^+^ cells represent a permanent record of embryonic KSP^+^ progenitors that have differentiated into other cell types and lost KSP^+^ cell identity.

To systematically analyze the persistent of KSP-lineage contribution to multiple nephron segments quantitative analyses were conducted at multiple time points spanning E19.5, P1, P30, P90, and P180 (Figures 3, 4, Supplementary Figures S3-S5). The results indicate that the proportion of RFP^+^ cells in each nephron segment increased progressively (Supplementary Tables S3-S7), indicating that KSP^+^ cell differentiation continues throughout kidney development. Notably, because these analyzes are based on a constitutive lineage-tracing system, the observed increase in the proportion of KSP-derived (KSP^−^RFP^+^) cells within a given segment reflects cumulative lineage contribution (including proliferation and survival) rather than direct measurement of *de novo* differentiation events per segment. Comparison of the proportions of KSP-derived cells in all nephron segments at different developmental stages revealed that KSP^+^ cells preferentially participate in postnatal kidney development. These findings established that KSP^+^ cells after E14.5 are not terminally differentiated but instead function as progenitors with broad multipotent differentiation potential, with their differentiation range far exceeding the limited distribution pattern of KSP^+^ cells in mature kidneys. While the current data cannot completely rule out the alternative interpretation that KSP^−^RFP^+^ cells merely represent the downregulation of KSP protein in mature differentiated cells rather than true progenitor differentiation, and definitively confirming the stage-specific progenitor origin remains to be addressed in future studies utilizing inducible *Ksp-CreER* lineage tracing systems, inherent limitations of this constitutive Cre system further require verification using dual recombinase approaches to distinguish between these possibilities. These results complement existing knowledge regarding the early role of Six2^+^ progenitors in nephrogenesis (Self et al., 2006). While Six2^+^ progenitors dominate early nephrogenesis (beginning E10.5) (Kobayashi et al., 2008), our findings position KSP^+^ cells as a complementary population that primarily functions during postnatal tubular maturation (after E14.5). Consistent with a previous report (Thomson et al., 2021), Ksp-deficient mice exhibit postnatal urinary concentrating defects, suggesting that KSP^+^ cells possess regulatory functions in collecting duct morphogenesis beyond structural adhesion. Our study further extends this functional observation to the cellular level, indicating through continuous differentiation their contribution to nephron formation. Furthermore, while previous studies have suggested the potential involvement of KSP^+^ cells in late development (Shao et al., 2002a; Thomson et al., 2021; Wertz and Herrmann, 1999; Bai et al., 2002), they lacked quantitative evidence and failed to capture this distinct temporal dynamic—where differentiation is minimal during embryogenesis (E19.5) but surges during postnatal stages to form entire tubules.

These developmental findings raise a critical question: do KSP^+^ cells in adult kidneys possess the properties of progenitor cells, or do they merely represent markers of mature epithelial cells? To address this question, we systematically analyzed the characteristics of KSP^+^ cells under homeostatic and injury conditions. The results revealed that KSP^+^ cells rarely proliferate under steady-state conditions, with CldU^+^, Ki67^+^, and PCNA^+^ cells accounting for only 0.05%, 0.31%, and 0.25% of KSP^+^ cells, respectively (Figures 5B,C, Supplementary Figure S6), indicating a quiescent state. This characteristic resembles that of AQP2^+^B1^+^B2^+^ progenitors reported by Gao et al. (Gao et al., 2022), yet falls below the basal proliferation rate of Pax2^+^ progenitors documented by Lazzeri et al. (Lazzeri et al., 2018), possibly reflecting functional specialization among distinct progenitor populations. Unlike the AQP2^+^B1^+^B2^+^ progenitors, which are primarily restricted to the distal tubules and collecting ducts, KSP^+^ cells represent a distinct progenitor population with a broader differentiation spectrum spanning multiple nephron segments (Gao et al., 2021). The CldU labeling system employed herein excludes false-positive signals resulting from apoptosis or necrosis (Bradford and Clarke, 2011; Humphreys et al., 2011). Manual counting under microscopy confirmed high concordance between S-phase cell proportions and CldU positivity, validating the reliability of proliferation data. Whereas previous studies have focused predominantly on acute proliferation during the first 3 days post-injury (Li et al., 2025), our long-term tracking reveals that KSP^+^ cell proliferation peaks extend to day 14. Furthermore, our quantitative assessment of segment-specific lineage contributions (Supplementary Figures S7-S10, Supplementary Tables S12-S15) highlights the dynamic and heterogeneous responses of KSP^+^ cells across different nephron segments during injury progression. While KSP^+^ cells broadly participate in early repair across multiple segments (D2-D6), their reparative contribution becomes segment-specific at the late fibrotic stage (D14), with sustained engagement in Megalin^+^ proximal tubules and Umod^+^ thick ascending limbs, but diminished contribution to AQP1^+^ and AQP2^+^ cells, likely reflecting differential segment-specific vulnerabilities to severe obstructive injury. In our UUO model, this proliferative phase coincides with the classic progression of renal fibrosis, which is characterized by sustained TGF-β1/Smad3 activation and subsequent collagen deposition (Liu et al., 2026; Zhang et al., 2024). While this temporal synchronization suggests a potential link between KSP^+^ cell dynamics and fibrotic progression, whether KSP^+^ cells directly drive fibrosis or merely respond to the UUO-induced microenvironment remains to be functionally validated.

This temporal pattern indicates that KSP^+^ cells represent among the earliest mobilized and proliferatively expanded cell populations following injury, directly participating in the injury response. These findings support the “dedicated progenitor-mediated” repair model (Humphreys et al., 2011; Kusaba et al., 2014), resonating with studies by Gao et al. on AQP2^+^ progenitors (Gao et al., 2020) and collectively establishing the existence of functional progenitor reserves in adult kidneys. Given the central role of Notch signaling in kidney development and stem/progenitor cell regulation across multiple tissues (Mukherjee et al., 2019b; Bray, 2016), we further investigated dynamic changes in this pathway in the UUO model to explore its correlation with the injury-specific activation of KSP^+^ cells. We observed that the protein levels of Notch1 and its ligand JAG2 were upregulated post-injury (Figures 6B,C), with the proportion of downstream effector Hes1 in KSP^+^ cells increasing from 0.21% to 14.55% by day 14 (Figure 6A), highly synchronized with KSP^+^ cell proliferation dynamics (Figure 6D). While this tight temporal synchronization suggests a potential link between KSP^+^ cell dynamics and Notch activation, it is important to note that correlation analysis does not establish causality. Functional validation of the Notch pathway in KSP^+^ cells represents a priority for future research. However, studies have demonstrated that Notch signaling exhibits cell-type-specific functions in the kidney (Mukherjee et al., 2019b), and the present study did not distinguish the differentiation tendencies of KSP^+^ cells toward distinct progeny; whether Notch activation biases specific cell fates remains unclear. Future studies could explore this potential causal axis through systemic gamma-secretase inhibitor (e.g., DAPT or LY-3039478) administration to test whether global Notch blockade blunts injury-triggered KSP^+^ cell proliferation, while generating Ksp-Cre-driven Notch1 or JAG2 conditional knockout mice would provide deeper insights into its cell-autonomous role in reparative phenotypes post-UUO.

Our findings exhibit both consistencies and important differences compared with existing progenitor studies. Relative to Sox9^+^, Protrudin^+^, or Pax2^+^ cell populations (Lazzeri et al., 2018; Oliver et al., 2016; Kang et al., 2016), the uniqueness of KSP^+^ cells lies in their origin from early embryonic development (E14.5) and their capacity to differentiate into epithelial cells across multiple nephron segments. Through the construction of the *Ksp-Cre*: *RFP* ^*f/f*^ lineage-tracing mouse model combined with validation across multiple time points from E14.5 to P180, this study demonstrates that although RFP^+^ cells in adult kidneys partially lose KSP expression, they originate embryonically from E14.5 KSP^+^ cells and continuously differentiate into all nephron segments (PTs/LHs/DCTs/CDs), thereby overcoming the limitation of traditional constitutive *Ksp-Cre* systems in distinguishing quiescent progenitors from dedifferentiated mature epithelium. (Kusaba and Humphreys, 2014). Finally, it should be noted that the possibility of distinguishing progenitors from dedifferentiated epithelium remains an open question for future works. The traditional view holds that tubular repair depends primarily on proliferation of surviving epithelial cells (Li et al., 2024), a model sufficient for acute injury but leading to cellular senescence and fibrosis in chronic or repetitive injury (Ferenbach and Bonventre, 2015). Our study, together with recent work, establishes the existence of functional progenitor reserves in adult kidneys (Rinkevich et al., 2014), whose activation failure may constitute a critical factor in repair failure. Nevertheless, single-cell transcriptomic analyses reveal substantial heterogeneity among kidney epithelial cells (Park et al., 2018; Wu et al., 2018); whether KSP^+^ cells comprise functional subpopulations and how these correspond to specific clusters in single-cell atlases require future investigation utilizing FACS-based isolation or single-cell RNA sequencing of KSP^+^ cells before and after injury.

In summary, this study identifies a population of KSP^+^ cells that functions as renal progenitor cells through *in vivo* lineage tracing. KSP^+^ cells are characterized by embryonic origin, multipotent differentiation potential, and injury-specific activation, revealing that they act as core drivers of postnatal renal patterning and are associated with Notch signaling during the injury response. These findings deepen our understanding of endogenous kidney repair mechanisms and provide experimental evidence for developing regenerative therapeutic strategies targeting specific cell populations or signaling pathways. Importantly, however, the possibility of distinguishing genuine progenitors from dedifferentiated epithelium remains an open question for future work, and addressing this issue will be essential for a more precise understanding of the cellular dynamics underlying kidney repair.

## 6 Significance statement

The role of KSP^+^ cells in kidney development and injury repair remains unclear. Here, we present *in vivo* lineage tracing evidence indicating that KSP^+^ lineage behaves as a multipotent progenitor population and participate in kidney development, particularly during the postnatal period. Consistent with our embryonic staining results and published reports (Shao et al., 2002a; Igarashi et al., 1999), KSP^+^ cells first emerge at embryonic day 14.5 (E14.5) during kidney development and are capable of differentiating into cells of multiple nephron tubule segments, from the proximal tubule to the collecting duct. During embryonic development, KSP^+^ cells exhibit limited differentiation capacity, with KSP-derived cells sporadically distributed across nephron tubule segments. In contrast, during postnatal tubular maturation and remodeling in mice, KSP^+^ cells exhibit robust differentiation potential and contribute to the formation of entire renal tubules across multiple nephron segments. Given the well-established role of Notch signaling in regulating renal progenitor activation and tissue repair, KSP^+^ cells in the adult kidney display active proliferation potential following injury, which is associated with Notch activation. These findings deepen our mechanistic understanding of endogenous renal progenitor biology during kidney development and injury, and provide new clues for kidney regenerative medicine.

## Data Availability

The original contributions presented in the study are included in the article/Supplementary Material, further inquiries can be directed to the corresponding author.
