# Supplementary material for: Lineage tracing reveals KSP+ cells as multipotent progenitors driving postnatal renal development and injury repair via notch signaling
Source: Front Cell Dev Biol. 2026 Sep 9;14:1893353. doi: 10.3389/fcell.2026.1893353 (PMC13597843; doi:10.3389/fcell.2026.1893353)
Supplement: Supplementary file 1 [file DataSheet1.docx]

**Supplementary Figures and Tables**

**Figure S1. Genotype identification of** ***Ksp-Cre: RFP^f/f^* mice.**

Lanes were labeled with primer pairs specific for the *Ksp-Cre* transgene (mutant-type: 420 bp; wild-type: 335 bp) and the *Rosa-LSL-tdTomato* (RFP) allele (mutant-type: 196 bp; wild-type: 297 bp). Primer sequences are listed in Table S2. All experimental mice used in this study were of the *Ksp-Cre^f/+^: RFP^f/f^* (*Ksp-Cre: RFP^f/f^*) genotype (hereafter designated as *RFP^KC^*). WT, wild-type; MT, mutant-type.

**Figure S2. Developmental tracing of KSP⁺ cells in *RFP^KC^* mice from E10.5 to E16.5.**

A-G. Immunofluorescence staining of KSP (green) and RFP (red) in embryonic kidneys at E10.5, E11.5, E12.5, E13.5, E14.5, E15.5, and E16.5, respectively. No KSP⁺ signal was detected before E13.5 (A-D). At E14.5 (E), KSP⁺ cells were first observed, and all RFP⁺ cells were exclusively KSP⁺, indicating faithful labeling of KSP⁺ cells by the RFP reporter. At E15.5 (F) and E16.5 (G), KSP⁺RFP⁺ double-positive tubular cells progressively increased. White arrows indicate KSP⁺RFP⁺ cells. Scale bars, 50 μm. n=4. 4-5 fields were analyzed per mouse.

**Figure S3. Lineage tracing of KSP⁺ cells at P1 in *RFP^KC^* mice.**

A. An overview of P1 kidney showing extensive expression of KSP (green) and RFP (red) in developing nephrons. DAPI (blue) marks nuclei. Arrows indicated representative tubules in B-C. B-C. Representative RFP^+^ tubule lost the expression of KSP. Scale bars, 50 µm (A); 200 µm (B-C). D-F. Triple immunofluorescence staining showing KSP^-^RFP^+^ tubules gained the expression of different segment-specific markers at P1. D. AQP2 for collecting duct tubule (arrow). E. Megalin for proximal tubule (arrow). F. Umod for thick ascending limb tubule (arrow). G.AQP1 for proximal tubule/descending thin limb tubule (arrow).Scale bars, 30 µm (D-F), 20 µm (G). n=4. 4-5 fields were analyzed per mouse.

**Figure S4. Lineage tracing of KSP⁺ cells at P30 in *RFP^KC^* mice.**

Triple immunofluorescence staining showing KSP^-^RFP^+^ tubules gained the expression of different segment-specific markers at P30. A. AQP2 for collecting duct tubule (arrow). B. Megalin for proximal tubule (arrow). C. AQP1 for proximal tubule/descending thin limb tubule (arrow). D. Calbindin for distal convoluted tubule (arrow). Scale bars, 20 µm (A-D). n=4. 4-5 fields were analyzed per mouse.

**Figure S5. Lineage tracing of KSP⁺ cells at P180 in *RFP^KC^* mice.**

Triple immunofluorescence staining showing KSP^-^RFP^+^ tubules gained the expression of different segment-specific markers at P180. A. AQP2 for collecting duct tubule (arrow). B. Calbindin for distal convoluted tubule (arrow). C. Megalin for proximal tubule (arrow). D. Umod for thick ascending limb tubule (arrow). Scale bars, 10 µm. n=4. 4-5 fields were analyzed per mouse.

**Figure S6. Dynamic changes in PCNA expression in KSP^+^ cells following UUO injury.**

Triple immunofluorescence staining of KSP (green), PCNA (red), and DAPI (blue) in WT mice kidneys at D0 (sham), D2, D6, and D14 post-UUO. A. D0: Rare PCNA⁺KSP⁺ cells (arrow) indicating early proliferative activation. B. D2: Rare PCNA⁺KSP⁺ cells (arrow) indicating early proliferative activation. C. D6: Increased PCNA⁺KSP⁺ cells (arrow) with ongoing DNA replication. D. D14: Sustained PCNA expression in KSP⁺ cells (arrow) during repair progression. Scale bars, 30 µm (A-B)；40 µm (C-D). n=4. 4-5 fields were analyzed per mouse.

**Figure S7. Lineage tracing in UUO model of X^+^RFP^+^KSP^-^ cells at D0 in *RFP^KC^* mice.**

Triple immunofluorescence staining showing KSP^-^RFP^+^ tubules gained the expression of different segment-specific markers at D0. A. AQP1 for proximal tubule/descending thin limb tubule (arrow). B. Megalin for proximal tubule (arrow). C. AQP2 for collecting duct tubule (arrow). D. Umod for thick ascending limb tubule (arrow). Scale bars, 20 µm (A-D). n=4. 200 tubules were quantified per mouse across 5 fields.

**Figure S8. Lineage tracing in UUO model of X^+^RFP^+^KSP^-^ cells at D2 in *RFP^KC^* mice.**

Triple immunofluorescence staining showing KSP^-^RFP^+^ tubules gained the expression of different segment-specific markers at D2. A. AQP1 for proximal tubule/descending thin limb tubule (arrow). B. Megalin for proximal tubule (arrow). C. AQP2 for collecting duct tubule (arrow). D. Umod for thick ascending limb tubule (arrow). Scale bars, 20 µm (A-D). n=4. 200 tubules were quantified per mouse across 5 fields.

**Figure S9. Lineage tracing in UUO model of X^+^RFP^+^KSP^-^ cells at D6 in *RFP^KC^* mice.**

Triple immunofluorescence staining showing KSP^-^RFP^+^ tubules gained the expression of different segment-specific markers at D6. A. AQP1 for proximal tubule/descending thin limb tubule (arrow). B. Megalin for proximal tubule (arrow). C. AQP2 for collecting duct tubule (arrow). D. Umod for thick ascending limb tubule (arrow). Scale bars, 20 µm (A-D). n=4.

**Figure S10. Lineage tracing in UUO model of X^+^RFP^+^KSP^-^ cells at D14 in *RFP^KC^* mice.**

Triple immunofluorescence staining showing KSP^-^RFP^+^ tubules gained the expression of different segment-specific markers at D14. A. AQP1 for proximal tubule/descending thin limb tubule (arrow). B. Megalin for proximal tubule (arrow). C. AQP2 for collecting duct tubule (arrow). D. Umod for thick ascending limb tubule (arrow). Scale bars, 20 µm (A-D). n=4

**Table S1. List of abbreviations**

| **Abbreviation** | **Full Form** |
| --- | --- |
| AQP1 | Aquaporin 1 |
| AQP2 | Aquaporin 2 |
| BrdU | 5‑bromo-2’-deoxyuridine |
| BUN | Blood urea nitrogen |
| CDs | Collecting ducts |
| CKD | Chronic kidney disease |
| CNTs | Connecting Tubules |
| Cdh16 | Cadherin 16 |
| CldU | 5-chloro-2′-deoxyuridine |
| DBP | Diastolic blood pressure |
| DCTs | Distal convoluted tubules |
| Hes1 | Hairy and enhancer of split 1 |
| JAG2 | Jagged2 |
| KSP | Kidney-specific cadherin |
| Ki67 | Ki-67 antigen |
| MT | Mutant-type |
| Notch1 | Neurogenic locus notch homolog protein 1 |
| PCNA | Proliferating Cell Nuclear Antigen |
| PCR | Polymerase Chain Reaction |
| PTs | Proximal tubules |
| RFP | Red fluorescent protein |
| RFP^KC^ | Ksp-Cre: RFP^f/f^ |
| SBP | Systolic blood pressure |
| Umod | Uromodulin |
| UUO | Unilateral ureteral obstruction |
| WT | Wild-type |

**Table S2. PCR Primer Sequences**

| **Target Gene** | **Genotype** | **Primers** | **Sequence (5‘to3’)** | **Expected Size (bp)** |
| --- | --- | --- | --- | --- |
| **Ksp-Cre** | MT | Cdh16-Cre(Ksp1.3Cre) forward | GCAGATCTGGCTCTCCAAAG | 420 |
|  |  | Cdh16-Cre(Ksp1.3Cre) reverse | AGGCAAATTTTGGTGTACGG |  |
|  | WT | Cdh16-Cre(Ksp1.3Cre) forward | CATGCCAATGGTTCACTCTAAGGT | 335 |
|  |  | Cdh16-Cre(Ksp1.3Cre) reverse | TCTCTATGTCCCAAAGTGCAGACAC |  |
| **RFP** | MT | Rosa-LSL-tdTomato forward | GGCATTAAAGCA GCGTATCC | 196 |
|  |  | Rosa-LSL-tdTomato reverse | CTGTTCCTGTACGGCATGG |  |
|  | WT | Rosa-LSL-tdTomato forward | AAGGGAGCTGCAGTGGAGTA | 297 |
|  |  | Rosa-LSL-tdTomato reverse | CCGAAAATCTGTGGGAAGTC |  |

**Note**: Primers listed in this table were used for PCR-based genotyping of *RFP^KC^* mice. For RFP genotyping, mutant homozygotes (*RFP^f/f^*) display only the 196 bp band; heterozygotes display both 196 bp and 297 bp bands; wild-type mice display only the 297 bp band. For Ksp-Cre genotyping, transgenic-positive mice display the 420 bp MT band, whereas wild-type mice display the 335 bp WT band. Since homozygous Ksp-Cre mice are embryonic lethal, the transgene was maintained in the heterozygous state. MT, mutant-type; WT, wild-type.

**Table S3. Quantification of KSP^-^RFP^+^Megalin^+^ cells in all RFP^+^ cells and KSP^-^RFP^+^Megalin^+^ cells in all Megalin^+^ cells**

| Time | Total RFP^+^ | Total Megalin^+^ | KSP^-^RFP^+^ Megalin^+^ | KSP^-^RFP^+^ Megalin^+^/RFP^+^(%) | KSP^-^RFP^+^ Megalin^+^/Megalin^+^(%) |
| --- | --- | --- | --- | --- | --- |
| E19.5 | 1873 | 925 | 23 | 1.23 | 2.48 |
| D1 | 2910 | 1273 | 58 | 1.99 | 4.55 |
| D30 | 4600 | 1579 | 103 | 2.24 | 6.52 |
| D90 | 7626 | 3763 | 311 | 4.08 | 8.26 |
| D180 | 8979 | 4123 | 468 | 5.21 | 11.35 |

**Table S4. Quantification of KSP^-^RFP^+^AQP1^+^ cells in all RFP^+^ cells and KSP^-^RFP^+^AQP1^+^ cells in all AQP1^+^ cells**

| Time | Total RFP^+^ | Total AQP1^+^ | KSP^-^RFP^+^ AQP1^+^ | KSP^-^RFP^+^ AQP1^+^/RFP^+^ (%) | KSP^-^RFP^+^ AQP1^+^/AQP1^+^(%) |
| --- | --- | --- | --- | --- | --- |
| E19.5 | 1734 | 1527 | 23 | 1.32 | 1.51 |
| D1 | 2515 | 2273 | 45 | 1.79 | 1.98 |
| D30 | 4318 | 3879 | 201 | 4.65 | 5.18 |
| D90 | 6617 | 4239 | 414 | 6.25 | 9.76 |
| D180 | 9985 | 5906 | 786 | 7.87 | 13.31 |

**Table S5. Quantification of KSP^-^RFP^+^Umod^+^ cells in all RFP^+^ cells and KSP^-^RFP^+^Umod^+^ cells in all Umod^+^ cells**

| Time | Total RFP^+^ | Total Umod^+^ | KSP^-^RFP^+^ Umod^+^ | KSP^-^RFP^+^ Umod^+^/RFP^+^(%) | KSP^-^RFP^+^ Umod^+^/Umod^+^(%) |
| --- | --- | --- | --- | --- | --- |
| E19.5 | 1355 | 857 | 24 | 1.77 | 2.80 |
| D1 | 1615 | 1080 | 47 | 2.91 | 4.35 |
| D30 | 2319 | 1259 | 78 | 3.36 | 6.19 |
| D90 | 4341 | 2382 | 218 | 5.02 | 9.15 |
| D180 | 6179 | 3428 | 396 | 6.41 | 11.55 |

**Table S6. Quantification of KSP^-^RFP^+^Calbindin^+^ cells in all RFP^+^ cells and KSP^-^RFP^+^Calbindin^+^ cells in all Calbindin^+^ cells**

| Time | Total RFP^+^ | Total Calbindin^+^ | KSP^-^RFP^+^  Calbindin^+^ | KSP^-^RFP^+^ Calbindin^+^/RFP^+^(%) | KSP^-^RFP^+^ Calbindin^+^/Calbindin^+^(%) |
| --- | --- | --- | --- | --- | --- |
| E19.5 | 1877 | 658 | 29 | 1.54 | 4.41 |
| D1 | 2500 | 925 | 52 | 2.08 | 5.62 |
| D30 | 3895 | 1561 | 141 | 3.62 | 9.03 |
| D90 | 6524 | 2218 | 354 | 5.43 | 15.96 |
| D180 | 8320 | 2993 | 602 | 7.23 | 20.11 |

**Table S7. Quantification of KSP^-^RFP^+^AQP2^+^ cells in all RFP^+^ cells and KSP^-^RFP^+^AQP2^+^ cells in all AQP2^+^ cells**

| Time | Total RFP^+^ | Total AQP2^+^ | KSP^-^RFP^+^AQP2^+^ | KSP^-^RFP^+^ AQP2^+^/RFP^+^(%) | KSP^-^RFP^+^ AQP2^+^/AQP2^+^(%) |
| --- | --- | --- | --- | --- | --- |
| E19.5 | 1250 | 958 | 29 | 2.32 | 3.03 |
| D1 | 1640 | 1075 | 55 | 3.35 | 5.11 |
| D30 | 3700 | 2980 | 217 | 5.86 | 7.28 |
| D90 | 6674 | 5713 | 1192 | 17.86 | 20.86 |
| D180 | 10329 | 9016 | 2085 | 20.18 | 23.12 |

Note: Total KSP⁺ cells represent endogenous KSP-expressing cells; Total RFP⁺ cells represent lineage-traced KSP-derived cells. Segment-specific markers: Megalin (proximal tubule), AQP1 (proximal tubule and thin descending limb of Henle's loop), Umod (thick ascending limb of Henle's loop), Calbindin (distal convoluted tubule), AQP2 (collecting duct). KSP^-^RFP^+^X^+^/RFP^+^ (%) indicates the percentage of RFP⁺ cells that have lost KSP expression and gained X expression among total RFP⁺ cells, reflecting the differentiation efficiency of KSP⁺ cells toward nephron segment-specific cell types. Data represent cell counts from representative sections from 4 mice per group. E, Embryonic day; D, postnatal day.

**Table S8. Quantification of CldU labeling in KSP⁺ cells following UUO**

| Time | Total CldU^+^ | Total KSP^+^ | KSP^+^CldU^+^/KSP^+^(%) |
| --- | --- | --- | --- |
| D0 | 5 | 9577 | 0.05 |
| D2 | 7 | 10535 | 0.06 |
| D6 | 53 | 2438 | 2.17 |
| D14 | 95 | 1389 | 6.83 |

**Table S9. Quantification of Hes1 expression in KSP⁺ cells following UUO**

| Time | Total Hes1^+^ | Total KSP^+^ | KSP^+^Hes1^+^/KSP^+^(%) |
| --- | --- | --- | --- |
| D0 | 20 | 9530 | 0.21 |
| D2 | 25 | 9950 | 0.25 |
| D6 | 390 | 6151 | 6.34 |
| D14 | 252 | 1732 | 14.55 |

**Table S10. Quantification of Ki67 expression in KSP⁺ cells following UUO**

| Time | Total Ki67^+^ | Total KSP^+^ | KSP^+^Ki67^+^/KSP^+^(%) |
| --- | --- | --- | --- |
| D0 | 17 | 5440 | 0.31 |
| D2 | 23 | 6855 | 0.33 |
| D6 | 403 | 6434 | 6.26 |
| D14 | 245 | 1943 | 12.61 |

**Table S11. Quantification of PCNA expression in KSP⁺ cells following UUO**

| Time | Total PCNA^+^ | Total KSP^+^ | KSP^+^PCNA^+^/KSP^+^(%) |
| --- | --- | --- | --- |
| D0 | 13 | 5100 | 0.25 |
| D2 | 16 | 5832 | 0.27 |
| D6 | 178 | 4438 | 4.01 |
| D14 | 240 | 1865 | 12.87 |

Note: Proliferation markers: CldU (S-phase label), Ki67 (active cell cycle), PCNA (DNA replication). KSP^+^X^+^/KSP^+^(%) indicates the percentage of marker-positive cells among total KSP⁺ cells. Data represent cell counts from representative sections from 4 mice per group. D, day post-surgery; D0, sham-operated control; Ki67, marker of cells in G1/S/G2/M phases; PCNA, proliferating cell nuclear antigen; Hes1, hairy and enhancer of split 1 (Notch downstream effector).

**Table S12. Quantification of KSP^-^RFP^+^AQP1^+^ cells in all RFP^+^ cells and KSP^-^RFP^+^AQP1^+^ cells in all AQP1^+^ cells in UUO models**

| Time | Total RFP^+^ | Total AQP1^+^ | KSP^-^RFP^+^AQP1^+^ | KSP^-^RFP^+^ AQP1^+^/RFP^+^(%) | KSP^-^RFP^+^ AQP1^+^/AQP1^+^(%) |
| --- | --- | --- | --- | --- | --- |
| D0 | 2105 | 1239 | 135 | 6.41 | 10.89 |
| D2 | 1710 | 612 | 103 | 6.02 | 16.83 |
| D6 | 1520 | 403 | 88 | 5.78 | 19.85 |
| D14 | 906 | 210 | 31 | 3.42 | 14.76 |

**Table S13. Quantification of KSP^-^RFP^+^Megalin^+^ cells in all RFP^+^ cells and KSP^-^RFP^+^Megalin^+^ cells in all Megalin^+^ cells in UUO models**

| Time | Total RFP^+^ | Total Megalin^+^ | KSP^-^RFP^+^Megalin^+^ | KSP^-^RFP^+^ Megalin^+^/RFP^+^(%) | KSP^-^RFP^+^ Megalin^+^/Megalin^+^(%) |
| --- | --- | --- | --- | --- | --- |
| D0 | 2536 | 1210 | 105 | 4.14 | 8.67 |
| D2 | 2103 | 1065 | 131 | 6.23 | 12.30 |
| D6 | 1210 | 639 | 92 | 7.60 | 14.39 |
| D14 | 762 | 317 | 63 | 8.26 | 19.87 |

**Table S14. Quantification of KSP^-^RFP^+^AQP2^+^ cells in all RFP^+^ cells and KSP^-^RFP^+^AQP2^+^ cells in all AQP2^+^ cells in UUO models**

| Time | Total RFP^+^ | Total AQP2^+^ | KSP^-^RFP^+^AQP2^+^ | KSP^-^RFP^+^ AQP2^+^/RFP^+^(%) | KSP^-^RFP^+^ AQP2^+^/AQP2^+^(%) |
| --- | --- | --- | --- | --- | --- |
| D0 | 2258 | 1715 | 299 | 13.24 | 17.43 |
| D2 | 1913 | 325 | 90 | 4.70 | 27.69 |
| D6 | 1305 | 201 | 55 | 4.21 | 27.36 |
| D14 | 913 | 186 | 15 | 1.64 | 8.06 |

**Table S15. Quantification of KSP^-^RFP^+^Umod^+^ cells in all RFP^+^ cells and KSP^-^RFP^+^Umod^+^ cells in all Umod^+^ cells in UUO models**

| Time | Total RFP^+^ | Total Umod^+^ | KSP^-^RFP^+^Umod^+^ | KSP^-^RFP^+^ Umod^+^/RFP^+^(%) | KSP^-^RFP^+^ Umod^+^/Umod^+^(%) |
| --- | --- | --- | --- | --- | --- |
| D0 | 2308 | 1905 | 125 | 5.41 | 6.56 |
| D2 | 1655 | 563 | 108 | 6.52 | 19.18 |
| D6 | 926 | 345 | 95 | 10.25 | 27.53 |
| D14 | 617 | 226 | 64 | 10.37 | 28.31 |

Note: Total KSP^+^ cells represent endogenous KSP-expressing cells; Total RFP^+^ cells represent lineage-traced KSP-derived cells. Segment-specific markers: AQP1 (proximal tubule and thin descending limb of Henle's loop), Megalin (proximal tubule), AQP2 (collecting duct), Umod (thick ascending limb of Henle's loop). KSP^-^RFP^+^X^+^/RFP^+^ (%) indicates the percentage of RFP^+^ cells that have lost KSP expression and gained X expression among total RFP^+^ cells, reflecting the differentiation efficiency of KSP^+^ cells toward nephron segment-specific cell types. KSP^-^RFP^+^X^+^/X^+^ (%) indicates the percentage of KSP-derived cells among total segment-specific marker-positive cells. Data represent cell counts from representative sections from 4 mice per group. D, day post-surgery; D0, sham-operated control; UUO, unilateral ureteral obstruction.
